# Roquin exhibits opposing effects on RNA stem-loop stability through its two ROQ domain binding sites

**DOI:** 10.1073/pnas.2424434122

**Published:** 2025-04-09

**Authors:** Jan-Niklas Tants, Andreas Walbrun, Lucas Kollwitz, Katharina Friedrich, Matthias Rief, Andreas Schlundt

**Affiliations:** ^a^Institute for Molecular Biosciences and Biomolecular Resonance Center, Faculty of Biological Sciences, Goethe University Frankfurt, Frankfurt 60438, Germany; ^b^School of Natural Sciences, Department of Bioscience, Center for Functional Protein Assemblies, Technical University of Munich, Garching 85748, Germany; ^c^Institute of Biochemistry, Faculty of Mathematics and Natural Sciences, University of Greifswald, Greifswald 17489, Germany

**Keywords:** RNA folding, NMR, optical tweezers, Roquin, single-molecule

## Abstract

The interaction of mRNA and regulatory proteins is critical for posttranscriptional control. For proper function, these interactions, as well as the involved protein and RNA structures, are highly dynamic, and thus, mechanistic insights from structural biology are challenging to obtain. In this study, we employ a multifaceted approach combining single-molecule force spectroscopy (SMFS) with NMR spectroscopy to analyze the concerted interaction of the two RNA-binding interfaces (A-site and B-site) of the immunoregulatory protein Roquin’s ROQ domain with the 3’ untranslated region (UTR) of the *Ox40* mRNA. This 3’UTR contains two specific hairpin structures termed constitutive and alternative decay elements (CDE, ADE), which mediate mRNA degradation through Roquin binding. Our single-molecule experiments reveal that the CDE folds cooperatively, while ADE folding involves at least three on-pathway and three off-pathway intermediates. Using an integrated microfluidics setup, we extract binding kinetics to Roquin in real time. Supported by NMR data, we find opposing effects of the two Roquin subdomains on distinct regions of the ADE: While the A-site interacts strongly with the folded apical stem-loop, we find that the B-site has a distinct destabilizing effect on the central stem of the ADE owed to single-strand RNA binding. We propose that RNA-motif nature and Roquin A- and B-sites jointly steer mRNA decay with context-encoded specificity, and we suggest plasticity of stem structures as key determinant for Roquin–RNA complex formation. The unique combination of NMR and SMFS uncovers a mechanism of a dual-function RNA-binding domain, offering a model for target RNA recognition by Roquin.

## Significance

Local RNA structure is decisive for specific engagement with gene-regulatory proteins and, as a consequence, correct cellular function. However, its structure is often dynamic and, thus, challenging to study. Here, we show NMR and single-molecule force spectroscopy (SMFS) efficiently complement each other to provide high-resolution, time-resolved data on RNA folding intermediates during dynamic complex formation with the immune-regulating protein Roquin, which exploits multiple RNA-binding sites. Our data reveal a dual-mode binding of Roquin to RNA by firmly attaching to the stem-loop and, at the same time, destabilizing other regions, making them accessible to downstream interaction partners.

RNA *cis* elements contribute to the regulation of key cellular processes, including translational control. Often, cognate *trans*-acting factors, usually proteins, confer functionality to the underlying RNA–protein complexes. High specificity in RNA target recognition by proteins is key for a functional outcome, and both proteins and RNAs contribute to this (1). Proteins often achieve specificity in ribonucleoprotein (RNP) formation via their modular architecture or specialized RNA-binding domains, e.g. dsRBDs for double-stranded RNAs. Despite their limited tertiary contacts, RNAs exhibit a great structural variety. Due to inherent flexibility, RNA *cis* elements sample a large conformational space, and thus, one sequence can provide binding interfaces for more than one protein (2). Hence, such an RNA molecule visits many accessible structures and this conformational dynamics is key for its function (3), such as protein or small molecule binding. Ultimately, the spatiotemporal availability of a certain RNA structure will govern final *cis*–*trans* pair formation and affect downstream functions, which remains extremely difficult to predict and often not straightforward to analyze on a structural level (1).

The immune-regulatory protein Roquin is specialized for RNA hairpin recognition and mediates mRNA decay through *cis* element binding (4). The A-site located in its coreROQ domain interacts with single-stranded (ss) loops through base-specific contacts, while backbone contacts to apical stems assure shape recognition (5, 6). Additionally, structured extensions flanking the coreROQ domain and constituting the extended ROQ domain (extROQ) were suggested to interact with double-stranded RNAs through a B-site (7). Up to date, it remains elusive whether Roquin interacts with both types of RNAs simultaneously and whether both RNA structures are recognized within one *cis* element. An apparent affinity increase by binding both Roquin A- and B-sites suggested complex stabilization via the B-site binding to duplex regions distal to the hairpin (8). Roquin recognizes two types of hairpins, which contain a tri- or a hexaloop, named constitutive and alternative decay elements [CDE (5) and ADE (6), respectively] and were shown to be functionally redundant in the *Ox40* 3’UTR. Although both *cis* elements exploit the same Roquin binding interface, their overall shapes differ significantly (8, 9). While CDEs and ADEs are usually stable motifs (2, 8), Roquin does not require preformed stem-loop structures for initial engagement (2). Its capability to induce secondary structure and the structural variety of CDEs and ADEs challenge a simple model of combined recognition of hairpin and double-stranded regions through the Roquin A- and B-sites (9). Previous studies exploited only small isolated elements or a full 3’UTR context, which hampers sufficient probe coverage of individual *cis* elements due to its size. For Roquin and most other RBPs with multiple RNA-binding interfaces (10, 11), we thus currently lack detailed structural insights into the concerted interaction of all protein interfaces with target RNAs and a composite understanding of target specificity and context-related mRNA suppression.

As the example of Roquin shows, studying the structure and dynamics of large complexes of RNA and proteins generally poses a challenge for structural biology, and often, a single technique is not able to provide a complete picture. While NMR can provide detailed structural and kinetic information at the atomic level, larger structures are often elusive or not sufficiently accessible to NMR with the required resolution. Consequently, detecting and quantifying intermediates, especially in RNAs, remains challenging. Single-molecule force spectroscopy (SMFS) has given detailed insight into the dynamics of proteins and nucleic acids (12–14) ranging from microseconds to the timescale of minutes. A strength of SMFS is that it can be applied to large systems and still provide structural information on folding pathways and associated intermediates, at least on the level of pairwise distances between selected residues (15, 16), by steering the molecules through their folding free-energy landscapes. Even rare events populated to less than 0.01% can be detected (17). So far, studies on RNA structures have predominantly investigated dynamics and transition path times of folding (18–21). However, the interaction with cognate RNA-binding proteins, as is integral for Roquin-binding *cis*-elements, adds an additional layer of complexity to RNA dynamics and stability, a problem we sought to address in this study.

**Fig. 1.**
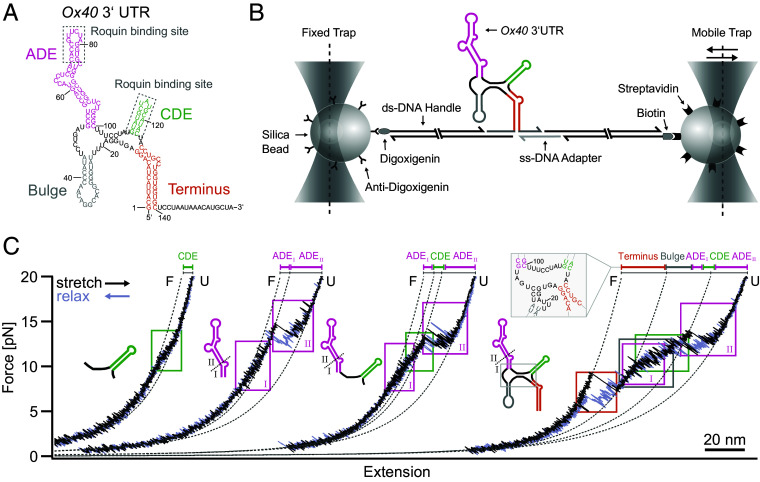
OT measurements capture the modular architecture of the *Ox40* 3’UTR. (*A*) Secondary structure of the fl *Ox40* 3’UTR as determined in (8). Individual structured/functional elements are color-coded. Note that this structure served as a starting point in this study and could not unambiguously determine potential secondary structures in the 3’UTR core region. (*B*) Setup and measurement principle of the dual-beam OT system used in this study (for details, *Materials and Methods*). (*C*) Representative force-extension curves of the isolated CDE, ADE, the combined ADE-CDE construct and the fl 3’UTR construct. Note that the extension axis does not display absolute values since the traces are arranged for side-by-side comparison. Unfolding/refolding transitions of structural elements are highlighted with boxes, and WLC fits to the most pronounced intermediates are shown as dashed lines. The unfolded contour length contribution of the Terminus obtained in the fl force-extension curves reveals an additional stemmed unit adjacent to the Bulge (gray inset). Traces are box-filtered to reduce the thermal noise (see *SI Appendix*, Table S1 for more details and *SI Appendix*, Fig. S1*B* as an example for a full-bandwidth trace). In *SI Appendix*, Fig. S2, we show a collection of five additional sample traces for each of the constructs used.

Here, we integrate high-resolution structural data from NMR with optical tweezers (OT), an SMFS technique, to monitor RNA–protein complex formation in real time. OT experiments confirm the overall fold of the full-length (fl) *Ox40* 3’UTR at nucleotide resolution (Fig. 1*A*), based on which we provide a detailed folding landscape of the extended *Ox40* ADE. While we observe stabilization of apical hairpins through the Roquin A-site, we give evidence for ssRNA binding through the B-site, leading to a destabilization of basal stem regions, using an interdisciplinary approach supported by bulk biochemical assays such as electrophoretic mobility shift assays (EMSA), CD spectroscopy, and hydroxyl radical probing. We precisely quantify conformational populations of ADE folding intermediates and observe a Roquin-induced shift in folding equilibrium. Our data show that Roquin contributes to the structural plasticity of RNAs by selectively exploiting its two RNA-binding sites. The binding of Roquin to ssRNAs partially explains the modulatory effect on RNA structure and further expands the known RNA target spectrum. This study serves as a paradigm on how SMFS and NMR spectroscopy supplemented with biophysical techniques provide time-resolved and quantifiable structural snapshots of RNA folding intermediates, important for and applicable to dynamic RNPs.

## Results

### OT Experiments Capture Structure and Folding Intermediates of the fl *Ox40* 3’UTR.

We initially employed an OT setup to study folding and unfolding dynamics of RNA structures within the *Ox40* 3’UTR. To this end, we designed a dumbbell construct in which a single RNA molecule is tethered between two beads, as illustrated in Fig. 1*B* [see *Material and Methods* and (22) for more details]. We investigated four different constructs with increasing complexity (8): the isolated CDE, the isolated ADE, a tandem ADE-CDE construct, and the fl *Ox40* 3’ UTR (Fig. 1*C*).

Fig. 1*C* shows representative examples of stretch (black) and relax (gray) cycles for each construct. The isolated CDE exhibits a rapid two-state folding/unfolding transition at 11.5 pN. Note that only the hump-like transition visible in the green box reflects CDE folding and unfolding, while the general convex shape of the trace results from the entropic elasticity of the DNA handles (23). Fits using the worm-like chain (WLC) model of polymer elasticity (24, 25) allow precise determination of the contour length gain during an unfolding event. We find an average length gain of 7.8 ± 0.2 nm (*SI Appendix*, Table S2) in agreement with the expected value of 15 bases transitioning from the folded to the fully extended state. While the smoothed trace filters out individual transitions, higher-bandwidth data in *SI Appendix*, Fig. S1 allow direct observation of folding/unfolding fluctuations happening on the sub-ms timescale.

For the isolated ADE, we find two prominent folding/unfolding transitions (Fig. 1*C*). A first transition (I) occurs at around 9 pN, followed by a more prominent transition (II) at 15 pN. Transition I is consistent with the unfolding of the basal stem until the first interior bulge G56-C93, while transition II subsequently involves the full unfolding of the remainder of the ADE. The length gains obtained by WLC fits (see *SI Appendix*, Table S2) fully agree with this interpretation.

Unfolding/refolding of the combined ADE-CDE construct displays features of both isolated elements, consistent with the structure in Fig. 1*A*. At low forces, we find transition I of ADE followed by the hump characteristic for CDE unfolding and finally, transition II of ADE unfolding as the last and most stable structural component. Again, the contour length gains determined by WLC fits match this interpretation (*SI Appendix*, Table S2). We find no indication of additional tertiary structural interactions between the two elements, supporting earlier findings that the two elements exist independently (8).

The fl *Ox40* 3’UTR construct exhibits two additional unfolding signatures corresponding to the Terminus and the Bulge. Based on the secondary structure predicted by *mfold* (26) (Fig. 1*A*), which was experimentally confirmed previously (8), one would expect the unfolding of the Terminus to either stop at the GGA bridge (nts 17 to 19) or to unfold even further in the same step, leaving only the CDE, ADE, and Bulge elements closed. Interestingly, the contour length gain extracted from the WLC fit (*SI Appendix*, Table S2) after the first unfolding transition (orange box) matches neither of these assumptions. This finding suggests that the GGA nucleotides (17–19) instead pair with UCC adjacently to the Bulge corresponding to the core region structure shown in the close-up of Fig. 1*C*. This structure is in line with the lowest free-energy structure predicted by *Vienna RNAfold* (27) (*SI Appendix*, Fig. S1*E*) (8), demonstrating the capability of SMFS in resolving ambiguities between RNA structure prediction tools.

Unfolding of the Terminus opens the structure so that all the remaining folded stem-loops are subject to force in parallel. Consequently, this leads to simultaneous folding/unfolding transitions of the remaining structural parts. We therefore find their individual signatures overlapping, making a strict separation of the individual transitions impossible, except for the ADE_II_ transition, which occurs last due to its higher mechanical and energetic stability (8). Notably, no additional folding event occurs when comparing individual elements with the fl 3’UTR. Interestingly, after complete unfolding and subsequent relaxation to zero force, the fl *Ox40* 3’UTR is often trapped in a misfolded state, likely due to a nonnative fold of the Terminus (*SI Appendix*, Fig. S2). Such behavior is well known for RNA constructs of similar complexity (28, 29), underscoring the critical role of controlled mRNA folding in its native context. Collectively, our OT experiments confirm the structural independence of the four *Ox40* 3’UTR elements and reveal that its global folding/unfolding network predominantly arises from the autonomous folding behavior of these elements. This qualifies the OT methodology for utilization in detailed interrogation of RNA-structural contexts.

### The *Ox40* ADE Samples Multiple Folding Intermediates.

We have previously shown that a CDE hairpin structure can exchange between a folded and unfolded state, both of which are recognized by individual RBPs (2). We thus asked whether a yet more pronounced intrinsic heterogeneity—affected by Roquin—is observed in the *Ox40* ADE, providing a more complex fold than the CDE.

**Fig. 2.**
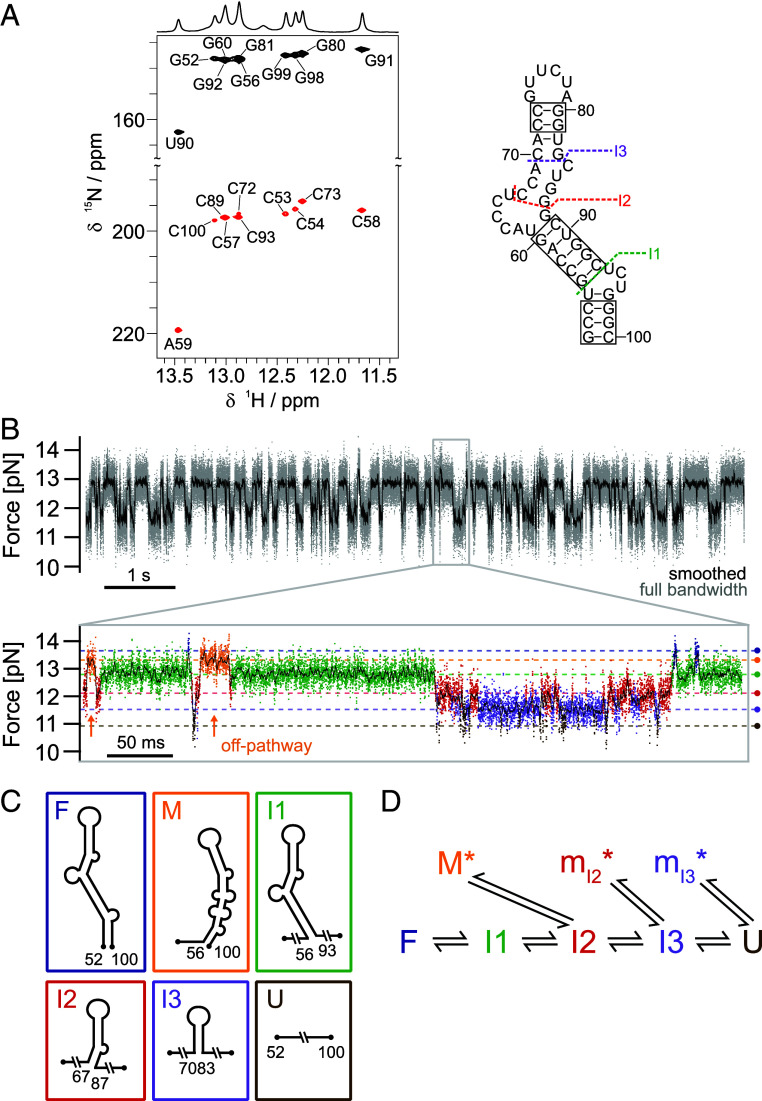
The *Ox40* ADE adopts a stable fold via multiple folding intermediates. (*A*) HNN-COSY experiment of apo ADE. H-bond donor peaks are shown in black, pairing H-bond acceptor peaks in red. Observed H-bonds confirm base pairs, as indicated in boxes on the ADE secondary structure on the *Right*. Dotted lines show the position of the intermediates extracted from OT experiments below. (*B*) Passive-mode measurement of the ADE. States are more folded toward higher forces and more unfolded at lower forces. Note that the folding process shortens the length of the construct, and hence, the beads are pulled out of the trap, increasing the load. The zoom into a 450 ms excerpt of this trace reveals six different states constituting the folding network, including three intermediates (green, red, and violet) and a misfolded state (orange). Appearances of the off-pathway misfold (orange) are highlighted with arrows to emphasize their exclusive accessibility from the red state. (*C*) Schematic depiction of the states forming the unfolding/refolding network of the ADE (see color code from *B*). The misfolded orange state is formed by non-native folding downstream of I2. The schematic structure M represents the most likely secondary structure based on its number of unfolded nucleotides, supported by predictions of the *RNAstructure fold* server (31) (see *SI Appendix*, Fig. S4*B* for more details). (*D*) Folding network of the ADE extracted from transitions observable in passive mode. While lifetimes of F, I1 and U are single-exponentially distributed, we see multiexponential distributions on the force levels of the purple, red, and orange states (*SI Appendix*, Fig. S4, ADE pathway), indicating ensembles of states with short-lived misfolds (indicated by an asterisk).

We first set out to determine the stability of individual ADE segments in the apo state and to capture its full conformational space. The high melting point of ~75 °C of the ADE described before (8) suggests formation of a network of stabilizing H-bonds. We recorded HNN-COSY (30) NMR spectra to detect and quantify base-pair-contained H-bonds (Fig. 2*A*). Indeed, we detected H-bonds for most base pairs described before (8), except for A71-U82 and U55-G97, confirming formation of strong duplex regions (Fig. 2*A*) interspersed with bulges. Increased temperatures led to destabilization of the majority of base pairs (*SI Appendix*, Fig. S3), as expected. However, the apical stem and the duplex region below the central bulge (residues 61 to 67) showed detectable imino proton signals at 37 °C, indicative of secondary structure. Base pairs of residues 67 to 70 with residues 83 and 85 to 87 were not directly observed, likely owed to the lower stability of this region. C84 and the adjacent central bulge render this stretch less stable, as indicated by the absence of imino proton signals (Fig. 2*A*). In sum, the NMR data support the outlined secondary structure scheme of the *Ox40* ADE on the level of individual base pair stabilities.

Next, we conducted OT experiments to gain more detailed insight into the folding free-energy landscape of the ADE and its unfolding/refolding pathway. In constant velocity experiments, the prominent intermediate described above is clearly visible (*SI Appendix*, Fig. S1*A*, I1, green WLC fit). However, a zoom-in of the trace reveals additional intermediates with contour lengths both above and below I1 (*SI Appendix*, Fig. S1*A*). To track folding/unfolding fluctuations over time and analyze the exchange kinetics between these various states, we then performed passive mode measurements (*Material and Methods*). We observed multiple close-to-equilibrium transitions between various intermediate states over many seconds (Fig. 2*B*). A zoom into this trace (Fig. 2*B*) allowed for observing the folding/unfolding pathway in real time. According to their different contour lengths, we identified six different levels. Based on those lengths, we ran a Hidden Markov Model [see *SI Appendix*, *Supplementary Methods* and (32) for more details] (HMM), which yielded an assignment of the data points to the various states (Fig. 2*B*) as well as the states’ lifetime distributions (*SI Appendix*, Fig. S4*A*). In sum, the ADE fluctuates between the fully folded state (F) and the completely unfolded state (U) (Fig. 2 *B* and *C*). While most states can directly pass to their neighboring ones, the orange state can only be populated from and depopulate into the red state, but we never observe a direct transition from the blue or green states into this state, or vice versa. This indicates that the orange state is an off-pathway intermediate, unable to productively fold further.

Hence, we identified the orange state (M) as a misfolded state, while all the other intermediates are on-pathway: I1 (green), I2 (red), and I3 (purple). From the force changes accompanying each transition, we calculated the associated gain in contour length (*SI Appendix*, Table S2) and suggest structural models for the respective intermediates (Fig. 2*C*). For the on-pathway intermediates, we assumed that each unfolding step disrupts a stem region along with its adjacent interior bulge. The resulting structures shown in Fig. 2*C* satisfy all constraints. For the misfolded intermediate (M), we used information about its contour length as well as its exclusive accessibility from I2 to infer its structural model. Free-energy differences at zero force between all the states assigned in passive mode are summarized in *SI Appendix*, Table S3 (see *SI Appendix*, *Supplementary Methods* for more details). From these free-energy values, it can be seen that, in the absence of force, the equilibrium strongly favors the folded state. Although partially folded intermediates are less populated and therefore beyond detectability in our NMR experiments, they are still important checkpoints in the folding free-energy landscape of the ADE.

Analysis of state lifetimes further suggests the presence of even more transient intermediates along the folding pathway. While the lifetime distributions of F, U, and I1 are single exponential, the distributions of I2, I3, and M are multiexponential (*SI Appendix*, Fig. S4*A*). This suggests that those states are, in fact, a mixture of different states occurring at similar contour lengths. We can, therefore, extend our model for the folding/unfolding pathway of ADE as shown schematically in Fig. 2*D*. While the on-pathway intermediates directly connect the native to the unfolded state, there are several branch points where misfolds can slow down the folding process. The misfold M could be directly identified due to its distinct length, while the other misfolded intermediates have a length indistinguishable from the intermediates I2 and I3. Hence, we denote them as m_I2_ and m_I3_. Note that, in principle, also parallel folding pathways could account for non-single-exponentiality of lifetime distributions. However, applying force favors folding pathways along a sequential zipping coordinate, thus making alternative pathways less likely. For this reason, we consider misfolds to be the more likely explanation. Suggestions for structural models of the misfolded intermediates are given in *SI Appendix*, Fig. S4*B*. In summary, the ADE samples a large conformational space, while its folding equilibrium is shifted toward the full stem-looped structure.

### Roquin Stabilizes the Apical Stem-Loop Structure of the *Ox40* ADE.

To assess the impact of Roquin on the ADE stability, we next studied the binding and unbinding of Roquin to the *Ox40* ADE in single-molecule experiments under near-physiological buffer conditions (low-salt). While the high-salt buffer used in previous experiments enhanced RNA stability through cation-mediated stabilization—enabling higher-resolution OT measurements—it was unsuitable for studying ROQ–RNA binding, which relies on electrostatic interactions.

**Fig. 3.**
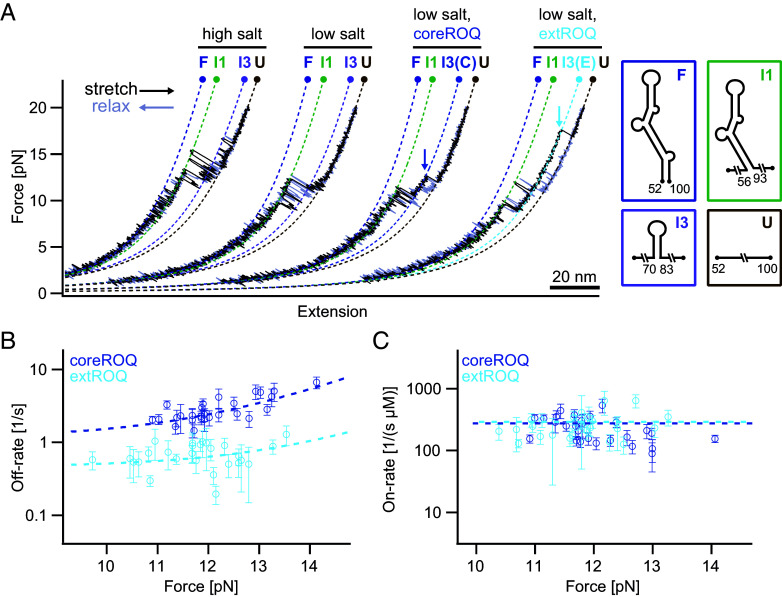
Roquin stabilizes the apical stem-loop of the *Ox40* ADE. (*A*) Representative force-extension curves of the ADE in different conditions: high salt (HS), low salt (LS), LS + coreROQ, LS + extROQ. Note that the extension axis does not display absolute values since the traces are arranged for side-by-side comparison. The measurements of the ADE with coreROQ and extROQ reveal stabilization of the same I3 intermediate (unbinding and unfolding events marked by dark blue (coreROQ) and light blue (extROQ) arrows) by both proteins. I3 (C) = coreROQ bound I3, I3 (E) = extROQ bound I3. (*B*) Unbinding rates of coreROQ (N = 27) and extROQ (N = 27) from ADE in LS buffer, extracted from passive-mode experiments and plotted against the force of the bound state in the respective traces. An exponential fit (dashed line, see *SI Appendix*, Table S4 for fitting parameters) was applied, assuming a force-dependent and a force-independent contribution to the off-rate. (*C*) Binding rates of coreROQ (N = 27) and extROQ (N = 27) to a folded ADE structure (see *Materials and Methods* for details), extracted from passive-mode experiments. A force-independent constant fit (see *SI Appendix*, Table S4 for fitting parameters, dashed line) was applied to the data points.

Utilizing our microfluidic chamber, which allowed exposition of the same individual molecule to up to three different buffer conditions (*SI Appendix*, Fig. S5), we confirmed that the essential features of the high-salt derived unfolding pathway were preserved in low-salt buffer (Fig. 3*A*). However, due to the reduced RNA stability in low-salt conditions, the resolution was lower, preventing clear identification of state I2, which we therefore omit from further annotation. The addition of coreROQ to the buffer resulted in distinct stabilization of I3, consistent with previously observed stabilization of CDEs (2), while extROQ binding led to an even higher stabilization of I3. Notably, both coreROQ and extROQ exclusively stabilize I3, representing the apical stem-loop structure of the *Ox40* ADE (base pairs made up of residues 70 to 73 and 80 to 83, Fig. 1*A*), but no other parts.

Analysis of binding kinetics in passive-mode experiments (*SI Appendix*, Figs. S6 and S7) showed that the increased stabilization by extROQ, compared to coreROQ, is driven by reduced off-rates, while on rates are identical (Fig. 3 *B* and *C*). The measured on-rates are not force-dependent (Fig. 3*C*), suggesting that the affinities of both coreROQ and extROQ to folded I3 are significantly higher than to unfolded RNA (see *Materials and Methods* for more details). From the measured on- and off-rates (*SI Appendix*, Table S4), we calculated equilibrium dissociation constants (K_D_) at zero force of 4.8 ± 0.4 nM (coreROQ) and 1.67 ± 0.23 nM (extROQ). For comparison, we performed a similar analysis for the CDE/Roquin interaction (*SI Appendix*, Fig. S8 and Table S4). We find on-rates are similar while off-rates are lower for extROQ, resulting in a K_D_ of 33 ± 2 nM (coreROQ) and 2.0 ± 0.4 nM (extROQ). These findings align with previous bulk data from (8), showing that the affinity of extROQ to CDE is comparable to extROQ/ADE in our single-molecule experiments. In summary, our results provide evidence for an RNA hairpin-stabilizing effect of Roquin, enhanced by lowered off-rates mediated through the Roquin B-site.

### Roquin Promotes Destabilization of Lower Stem Regions.

**Fig. 4.**
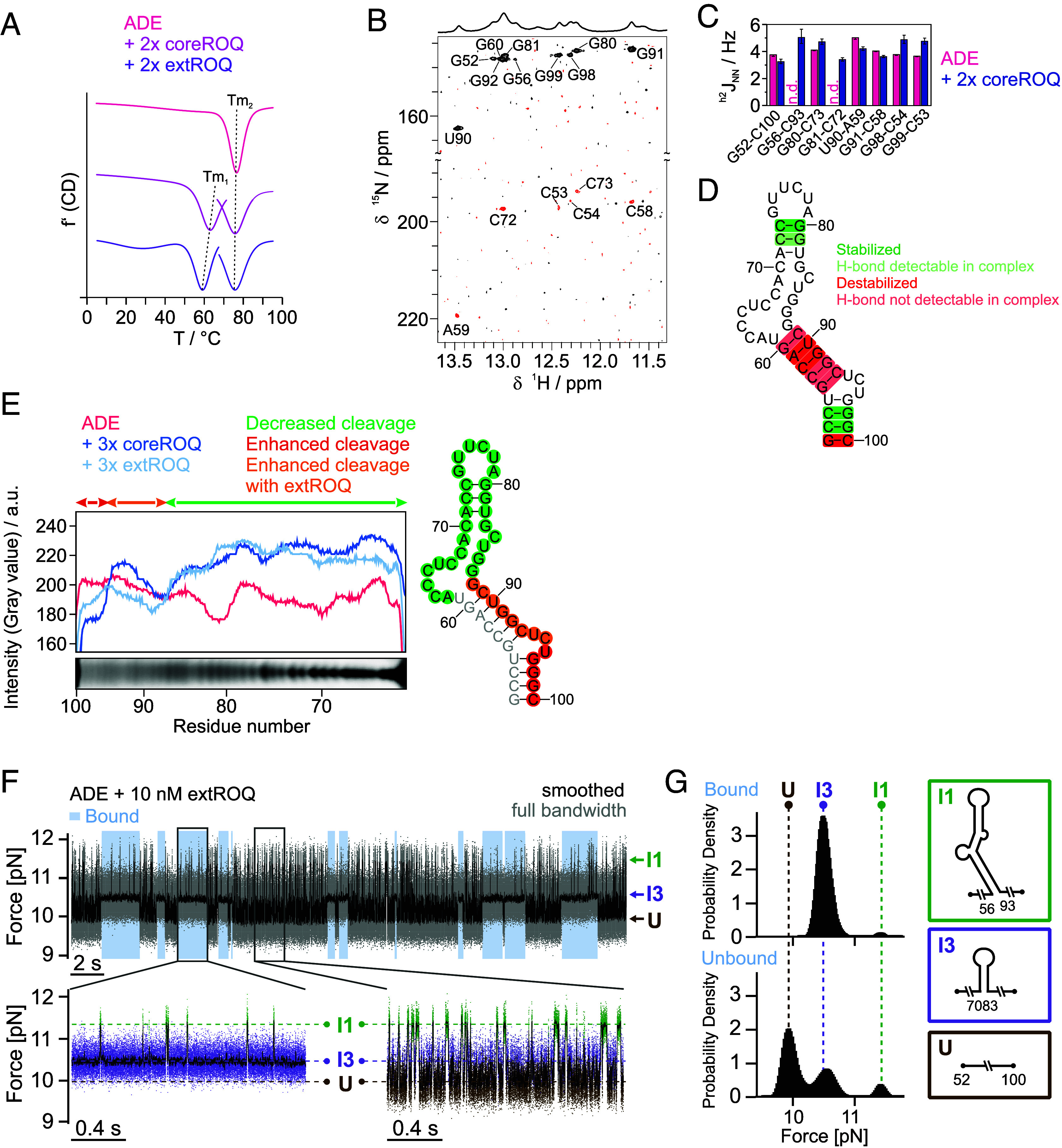
Roquin mediates destabilization of the lower *Ox40* ADE stem. (*A*) First derivative of CD melting curves of apo ADE and ADE bound to coreROQ and extROQ. Individual melting points are indicated by dashed lines. For RNPs the melting points were fitted individually. Note that the y-axis is not to scale and stacked for better comparison. (*B*) HNN-COSY spectrum of the ADE in complex with coreROQ. H-bond donor peaks are shown in black, pairing H-bond acceptor peaks in red. The corresponding 1D spectrum is shown above. (*C*) Quantification of H-bonds observed in HNN-COSY experiments of apo and complexed ADE shown in Fig. 2*A* and 4*B*. (*D*) Base pairs stabilized or destabilized by Roquin are highlighted in green and red, respectively. Faint green and red indicate bases that cannot be quantified in either of the two spectra. (*E*) Hydroxyl radical probing of apo ADE and ADE bound to coreROQ and extROQ. Cleavage profiles corresponding to the PAGE shown in *SI Appendix*, Fig. S9*E* are shown aligned to the alkaline ladder below. Colored arrows indicate regions with decreased or increased cleavage of the protein-bound ADE compared to the apo RNA. Differences in cleavage are plotted onto the ADE secondary structure on the *Right*. (*F*) *Top*, passive-mode trace of the ADE with 10 nM extROQ at a lower force regime where ADE populates folded structures up to I1. The protein-bound states (highlighted in light blue) can be discerned by strong stabilization of I3 and lower population of more folded structures. *Bottom*, representative zoom-ins to the bound (*Left*) and unbound (*Right*) phases. (*G*) Direct comparison of population probabilities in the extROQ bound (*Upper*) and unbound (*Lower*) case. The histograms display the distribution of the force data and show the different population probability of states U, I3 and I1 (indicated in dashed lines, corresponding structures on the *Right*), collected from the bound and unbound phases of the passive-mode trace shown in (*F*). Note that the second peak in the “Unbound” histogram is shifted to higher forces compared to the “Bound” histogram. This shift may reflect an equilibrium between I3 and I2 where I2 is more populated in the unbound state as compared to the bound state of extROQ indicating extROQ already destabilizes the I2 state.

As the ADE forms a structurally closed entity, the effect of Roquin onto the apical stem-loop and the basal structured segments needs to be assessed in the full ADE context. We first recorded CD melting curves of the ADE alone and in complex with core or extROQ (Fig. 4*A* and *SI Appendix*, Fig. S9). The apo ADE showed a melting point at 76.7 °C (T_m2_), in line with (8). Besides, we observed a less pronounced melting event at around ~65 °C (T_m1_), which could not be fitted. This initial transition, which was not observed before, is likely due to a local modulation of stability in the physiological buffer compared to (8), e.g. through the lack of stabilizing potassium ions. To our surprise, in the presence of the proteins we observed a decrease for both melting points. Strikingly, T_m1_ showed a stronger reduction than T_m2_, and the effect was more pronounced during binding of extROQ (*SI Appendix*, Fig. S9*A*). Roquin hence exhibits a destabilizing effect on parts of the RNA duplex, and the B-site actively contributes to this. This is also reflected in an overall decreased A-type helical content, observable by a lowered absolute starting signal of ellipticity in CD (33) (*SI Appendix*, Fig. S9*C*). Interestingly, the RNA–protein complex melting points are higher than the apo Roquin melting points (*SI Appendix*, Fig. S9*D*), suggesting a stabilizing effect of the RNA onto Roquin.

The CD melting data can only yield global readouts of regional stability, not considering nucleotide-resolved differences. Still, they suggest the existence of RNA regions with different responsiveness to Roquin binding. To track effects on the level of single base pairs, we recorded an HNN COSY NMR (30) spectrum of the coreROQ-bound ADE (Fig. 4*B*). While H-bonds were still found, the reduction in signal intensity, especially for H-bond acceptors, indicates an overall destabilizing effect of Roquin on the monitored duplex regions. We quantified the H-bonds of the apo and complex state (Fig. 4*C*) and observed an increase in H-bond strength for base pairs in the apical stem-loop and the basal stem (Fig. 4*D*), pointing at elevated stability. Interestingly, base pairs with reduced H-bond strength, and thus stability in presence of Roquin cluster in the stem region below the Bulge (bp 56 to 93 until bp 60 to 89, Fig. 4*D*). To corroborate our findings and to obtain information on non-base-paired residues, we performed hydroxyl radical probing (OH• probing) on the ADE in both its apo and Roquin-bound forms. As OH• probing reports on solvent accessibility of RNA, it can be used to track conformational changes within footprinting experiments (34). Cleavage profiles extracted from denaturing PAGE (*SI Appendix*, Fig. S9*E*) showed decreased cleavage of residues 62 to 87 in presence of either core or extROQ compared to the apo ADE (Fig. 4*E*). For residues 88 to 100, we observed increased cleavage, while the PAGE-limited resolution did not allow quantification of bands corresponding to residues 52 to 61. Upon mapping the changes in cleavage onto the secondary structure, we concluded that Roquin not only stabilizes the apical stem-loop but at least protects the adjacent stem structure and the Bulge from cleavage. In contrast, the entire structure below the Bulge is more solvent exposed and destabilized. Our NMR data and OH• probing experiments are thus in good agreement with CD melting curves and suggest that T_m1_ corresponds to melting of the lower ADE half. The upper half is stabilized through Roquin binding, likely accompanied by modulation of the RNA fold. This rationalizes the slight yet detectable decrease of T_m2_, while we locally observe stronger H-bonds in NMR. Hence, we claim that Roquin exhibits opposite effects on the stability of distinct stem regions.

### The Roquin B-Site Shifts RNA Structural Equilibria to Partially Opened Conformations.

To examine the influence of Roquin on the stability and folding kinetics of the individual ADE substructures, we next recorded passive-mode folding traces in the presence of extROQ at lower forces (Fig. 4*F* and *SI Appendix*, Figs. S10 and S11). In the full view (Fig. 4*F*), bound and unbound states can be clearly distinguished: While binding of extROQ has a pronounced stabilizing effect on I3 and prevents population of the unfolded state, the unbound states show rapid fluctuations between I3 and U. Besides the expected stabilization through extROQ binding, we observe that during bound phases, further folded states are less populated compared to unbound phases (i.e. fewer transitions from I3 to I1). As noted above, I2 and I3 cannot be reliably distinguished from each other at the low salt conditions and both populations are lumped into I3. Zoom-ins to bound and unbound phases (Fig. 4*F*, *Bottom*) clearly show this discrimination of the more folded structure (I1) relative to the less folded structure (I3) by extROQ. Fig. 4*G* directly compares the population distributions. The differences in population yield a destabilization of I1 by 1.3 ± 0.4 kcal mol^−1^ by interaction with extROQ. Analysis of folding/unfolding rates between I1 and I3 in the presence and absence of extROQ (*SI Appendix*, Fig. S11*F* and Table S5) show that extROQ predominantly slows the refolding rate while leaving the unfolding rate unaffected. Adding coreROQ also caused a destabilization of I1, albeit much smaller (0.34 ± 0.05 kcal mol^−1^, *SI Appendix*, Fig. S11*D*). Similar to extROQ, the effect of destabilization through coreROQ also acts on the folding rates rather than unfolding rates (*SI Appendix*, Fig. S11*E* and Table S5). Altogether, we propose a twofold effect of Roquin on the ADE structure: stabilization of the apical stem-loop by binding of the A-site and destabilization of the central stem by interaction of the B-site.

### The Roquin B-Site Binds ssRNAs.

**Fig. 5.**
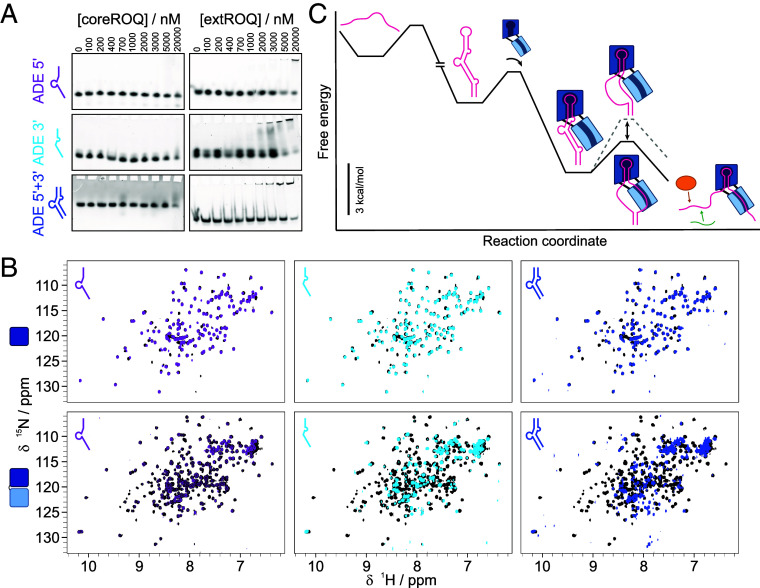
Roquin interacts with ss and ds RNAs through the B-site. (*A*) EMSAs of coreROQ and extROQ with ss ADE 5’ and 3’ halves and an annealed duplex. Protein concentrations are plotted on *Top*. (*B*) ^1^H,^15^N-HSQC spectra of apo (black) coreROQ (*Top*) and extROQ (*Bottom*) and in complex with the ss ADE 5’ half (purple), ss ADE 3’ half (cyan) and the ds ADE 5’ + 3’ of the central stem (blue). (*C*) Model of the energy landscape for extROQ binding the ADE. In a first step, ADE folds into its native structure. Next, extROQ engages with the apical stem-loop through its A-site. Binding of the B-site to ssRNA reduces the barrier for partial unwinding of the central stem regions by shifting the equilibrium toward a partially opened ADE. Destabilization of ADE stem structures facilitates *trans* factor engagement or enhances Roquin binding by conformational adjustments. The relative differences between energy minima are quantitative (assuming a 1 µM concentration of extROQ) while barrier heights are not.

A simple mechanism explaining the destabilization of the secondary structure between I3 and I1 through slowing of the refolding rate would entail interaction of parts of coreROQ and extROQ with single-stranded parts of the involved ADE region. We thus tested both proteins for interactions with the single-stranded halves of the ADE. We also included an RNA duplex annealed from the two halves, in order to probe for potential double-stranded (ds) RNA interactions as suggested by Tan et al. (7). Imino proton NMR spectra confirmed the single-stranded nature of the ADE halves as well as duplex formation for the annealed species (*SI Appendix*, Fig. S12). In EMSAs we did not observe binding of coreROQ to either of these RNAs (Fig. 5*A*), while extROQ interacted at low-micromolar concentrations with both single-stranded ADE halves and the duplex. This was confirmed by ^1^H,^15^N-HSQC NMR spectra (Fig. 5*B*): In the presence of ss or dsRNA, only minor chemical shift changes were observed for coreROQ (Fig. 5*B*, *Upper* row). ExtROQ, however, exhibited larger chemical shift perturbations (CSPs) and line-broadening for the single-stranded ADE halves and the duplex RNA (Fig. 5*B*, *Lower* row), confirming our EMSA results. Interestingly, in extROQ NMR experiments also signals assigned to the coreROQ domain were affected by RNA binding, suggesting that both A- and B-site contribute to recognition of ss and dsRNA, but that the B-site is the main driver of interaction. Roquin is thus able to not only interact with hairpin structures, but also recognizes ss and dsRNAs through its B-site.

We wondered whether sequence composition or geometry of the ADE stem affects RNP formation. To test this, we created an artificial ADE where we mirrored the stem, but maintained the loop orientation (*SI Appendix*, Fig. S13*A*, called ADE_mir_). Two mutations within the Bulge were required to stabilize the properly mirrored ADE geometry, and structural integrity was confirmed by a ^1^H,^1^H-NOESY NMR spectrum (*SI Appendix*, Fig. S13*A*). In EMSAs, both coreROQ and extROQ showed reduced binding to the ADE_mir_ (470 and 458 nM, respectively) compared to the wt ADE (307 and 322 nM, respectively) (*SI Appendix*, Figs. S13 *B* and *C* and S14). Smeary complex bands further suggested that the complex was less stable in a gel matrix. NMR ^1^H,^15^N-HSQC spectra of coreROQ with wt ADE and ADE_mir_ showed characteristic CSPs (*SI Appendix*, Fig. S13*D*) confirming complex formation, while extROQ complex spectra suffered from severe line broadening due to increased molecular weight and chemical exchange. Both coreROQ complex spectra were virtually identical (*SI Appendix*, Fig. S13*E*), suggesting a conserved ADE-like binding mode for the mirrored ADE version. We thus conclude that the mirrored ADE provides a less favorable geometry for RNP formation, resulting in lowered on-rates, but that Roquin induces an ADE-like fold for engagement, as suggested before (9).

## Discussion

The spatiotemporal availability of an RNA *cis* element’s functional conformation determines its engagement with cognate *trans*-acting binding partners, such as proteins. Despite its limited chemical space, RNA exhibits great structural heterogeneity and plasticity, also within RNPs. Small changes in RNA sequence, and hence structure, can significantly alter the RNA interactome, potentially leading to misfunctional and pathogenic RNPs. Consequently, we strive for a detailed understanding of RNP structures and mechanisms of complex formation as a basis for gene regulation or as the causative of diseases. However, detailed structural and mechanistic insights of plastic RNPs are challenging to obtain holistically, and available methods often fail to capture more than individual aspects in the full description of an RNP.

### The Combination of OT and NMR Captures and Quantifies RNA/RNP Folding Landscapes.

To address these challenges, we here combined NMR spectroscopy with single-molecule OT experiments and RNA–protein biochemistry to describe I) the folding landscape of a medium-sized RNA element, II) the specific interactions of two interrelated, nonredundant RBDs of the protein Roquin with distinct RNA element regions, and III) how Roquin exploits its multidomain architecture to modulate the RNA folding landscape. We integrated the high-resolution structural data from NMR with time-resolved single-molecule information from OT. We show that OT experiments provide single-nt information on a sub-ms timescale for RNAs varying from 29 nt to 140 nt (Figs. 1–4). NMR can help to assign folding events in OT traces. Conversely, the low amount of material required for OT experiments enables to test RNA folding under varying conditions to select promising features for en detail testing in NMR. The low concentrations used in OT also mitigate nonspecific intermolecular interactions, often observed for RNAs, based on stacking interactions (35, 36) or stem-loop intrinsic complementarity, even at small size (37).

Due to its inherent structural flexibility (8) the *Ox40* 3’UTR exhibits overlapping unfolding profiles, which we could reconstitute in a divide-and-conquer approach (Fig. 1*C*). OT experiments allowed to distinguish cooperative one-step folding/unfolding (CDE) from more complex multistep folding with multiple intermediate states found in ADE, ADE-CDE, and the fl construct. While NMR experiments confirmed a network of hydrogen bonds as the basis for a major stable conformation of the ADE (Fig. 2*A*), OT revealed a complex dynamic folding network with stable on and off-pathway intermediates (Fig. 2 *B*–*D*). Bulk measurements usually capture a population- and time-averaged conformation, whereas single-molecule experiments allow to dissect low-populated folding states more directly and with a much higher sensitivity. This now allowed to monitor unfolding of the ADE nts 67 to 69 (I2 in Fig. 2 *B* and *C*), a region previously invisible in NMR experiments (8), and to resolve discrepancies between two alternative structure predictions for the fl *Ox40* 3’UTR (Fig. 1*C*). The folding intermediates I3, I2, and I1, we find for ADE might assist correct cotranscriptional folding in vivo. These intermediates will likely start to form as soon as the polymerase reaches the apical stem-loop region of the ADE, locking in and protecting correctly folded parts against misfolding.

Multiple RNA folding intermediates and their constant exchange can provide target sites for multiple RBPs with varying specificities as shown before (2). The multiple intermediates we find for the *Ox40* ADE highlight the great plasticity RNAs can exhibit. How a single sequence can translate into heterogeneous structures could possibly explain inconsistencies between in vitro and in vivo data for RNA folds, as found by Andrzejewska et al. (38), and their susceptibility to fold-mediated interactions with RNA-binding proteins.

A valuable byproduct of OT measurements is quantitative information about thermodynamics and kinetics. In combination with microfluidics, we determined single-molecule affinities and on/off-rates for complex formation between ROQ and ADE as well as CDE. Our observed affinity of extROQ to ADE was significantly higher (~100-fold) compared to previously reported bulk measurements (8), obtained from EMSA (171 nM). Although useful for qualitative comparison, EMSAs often do not yield absolute affinities since they are carried out under nonequilibrium conditions. Here, single-molecule measurements offer an attractive alternative. The availability of kinetic data from this study can readily explain the enhanced affinity of ADE and CDE to extROQ vs. coreROQ as off-rate driven. Moreover, our results on the force dependence of the on-rates (Fig. 3*C* and *SI Appendix*, Fig. S8*C*) favor an interpretation where ROQ exclusively binds to already folded RNA substructures (conformational selection) but does not induce RNA structure by binding (induced fit).

We further showed that OT data can fully complement the gaps in ADE RNA coverage, found as technical limitations in NMR and biochemical probing (Fig. 4 *A*–*E*). For the Roquin–ADE complex, OT experiments not only agree well with NMR, CD, and structure probing data, but also enable the quantification of RNA conformational populations. We propose that this methodology is easily but efficiently adaptable to other highly specific RNA–protein complexes. Similar integrative approaches have already proven successful in other contexts. For instance, NMR combined with single molecule FRET has been used to measure protein/protein and protein/RNA binding kinetics (39, 40). Additionally, Jones et al. (41) employed NMR and OT to investigate microRNA structure and dynamics. While OT experiments in that study were limited to (un)folding of RNA, our combination of microfluidics-enhanced OT with NMR now reveals the energy landscape of protein/RNA interactions.

### Roquin Modulates RNA Structures in a Bimodal Fashion through A and B-Sites.

The experiments depicted in Fig. 4 suggest that, apart from the apical stem-loop, Roquin also interacts with other parts of the ADE. While this effect is small for coreROQ, binding of extROQ clearly destabilizes the ADE structure basal to the apical stem-loop. Our integrated approach uniquely identifies the affected region as the central stem between residues 56 to 60. How can we explain the B-site-induced destabilization of the central stem?

In summary, our data support the sequence of events outlined in Fig. 5*C*. After initial folding, extROQ binds strongly to the apical stem-loop via its A-site. Even though we found that the Roquin B-site can bind both ss and dsRNA with a similar, low µM-affinity (Fig. 5*A*) the observed reduction in central stem stability suggests that extROQ favors binding to ssRNA for efficient engagement with the full ADE. This preference could be supported by structural constraints occurring with the A-site tightly bound to the apical stem-loop and the B-site trying to engage with the adjacent central stem. In this scenario, unfolded flexible ssRNA strands, rather than duplexed RNA, may facilitate proper engagement. Note that the conformation of an A-site-only engagement is thermodynamically favored over the fully engaged conformation (Fig. 5*C*) as the energetic cost of opening the central stem (*SI Appendix*, Table S3) outweighs the free energy gained from B-site binding (see Fig. 4*G*). However, B-site engagement with ssRNA facilitates the opening of the central stem necessary for the binding of downstream effectors. It both reduces the kinetic barrier for the separation of the two RNA strands and shifts the conformational equilibrium of the central stem toward unfolded (Fig. 5*C*). In this context, the interior bulge involving nt 62 to 66 may act as a nucleation point to initiate ssRNA binding, thus facilitating splitting of the double strand. Unpaired regions have previously been shown to couple and modulate dynamics among neighbored structured regions (42). The exact mechanism remains to be explored, but our EMSAs suggest a moderate preference for the ADE 3’ strand engaged by the B-site.

The role for the B-site in Roquin as a ssRNA binder expands its target spectrum beyond hairpins and dsRNA. In line with previous findings, the ssRNA binding capacity of Roquin might have served in target selection during evolution (9) and be of particular relevance in unstable element binding. In this, the ROQ domain B-site could have enhanced affinity to otherwise less favorable targets, i.e. with unstable hairpins or stem-loops of suboptimal geometry. Thereby the rather promiscuous binding of the B-site potentially compensated for weaker A-site binding.

As ADEs differ significantly from CDEs in structure (9) and show a distinct folding behavior (this study) we speculate that structural features like the central ADE Bulge are required for modulation by Roquin also in other targets, i.e. to initiate unwinding. Possibly, Roquin modulates RNA *cis* element stability to reach an optimal window for subsequent engagement. At the same time, this mechanism exposes the newly formed, now unpaired region of the stem for possible interactions with *trans* factors, be it microRNAs or proteins, that could contribute to regulation by Roquin (Fig. 5*C*), e.g. in acting as target sites for recruited nucleases.

In the context of posttranscriptional regulation by Roquin, we suggest that specific target mRNA recognition and suppression additionally involve RNA sequence-encoded dynamics and shift-in-equilibrium caused by the protein. Those remain challenging to put into numerical descriptors. But it appears obvious that a canonical target sequence of Roquin, besides the previously described stem-loops recognized by coreROQ [including recent specification of loop requirements (43), needs to take into account the differential affinity of the extROQ B-site to an adjacent RNA region as well as its intrinsic plasticity and response to ROQ-binding. Furthermore, a comprehensive understanding of Roquin-mediated gene regulation will require elucidating its interactions with other proteins (44, 45), and potential Roquin cooperativity or homodimerization exploiting the occurrence of multiple *cis* elements in a 3’UTR (46).

In summary, our integrated approach enabled precise detection and quantification of folding intermediates in a dynamic RNA, both alone and in complex with a two-domain RBP. We provided mechanistic insights into how two RBDs exert opposing effects on RNA structure stability and plasticity to fine-tune complex formation, representing a key determinant of specificity.

## Material and Methods

### Protein Expression and Purification.

The ROQ domain of murine Roquin-1 was expressed as two constructs as described before (8): coreROQ and extROQ were expressed in *Escherichia coli* BL21 at 37 °C overnight while shaking. For isotope labeling minimal M9 medium was used supplemented with ^15^NH_4_Cl. Briefly, cells were harvested for 20 min at 4,000 rpm and 4 °C and resuspended in 500 mM NaCl, 50 mM Tris pH 8.0 and 3 mM β-Mercaptoethanol, supplemented with protease inhibitor (Protease Inhibitor Mix G from SERVA, Heidelberg, Germany) and lysozyme. After sonication proteins were purified via IMAC (immobilized metal ion affinity chromatography; Ni^2+^ resin obtained from Roth, Karlsruhe, Germany) in the same buffer containing imidazole concentrations of 30, 100, and 500 mM. After TEV protease (tobacco etch virus; made in-house) cleavage and dialysis against imidazole-free buffer at 4 °C overnight, proteins were passed through a reverse IMAC. The flow-through was concentrated and purified via SEC (Superdex S75 16/600 from GE) in 1 M NaCl, 50 mM Tris pH 7.0 and 2 mM TCEP. Proteins were buffer exchanged to 150 mM NaCl, 50 mM Tris pH 7.0 and 1 mM TCEP in an Amicon® centrifugal filter unit with a molecular weight cut-off of 10 kDa. Purity and structural integrity of the proteins were checked by SDS-PAGE and NMR spectroscopy.

### RNA In Vitro Transcription and Purification.

Templates for in vitro transcription of RNAs for OT experiments and mirrored ADE were generated by PCR and Gibson assembly of target sequences into HDV-containing vector (Hepatitis D virus ribozyme). Wt CDE, ADE tandem ADE-CDE, and fl *Ox40* 3’UTR constructs were already available from (8). RNAs were transcribed and purified as before (8), i.e. after transcription with 400 nM homemade T7 polymerase for 3 h at 37 °C (4 mM of each NTP, 50 mM Tris-HCl pH 7.5, 40 mM MgCl_2_, 5 mM DTT, 2 mM Spermidine) RNAs were precipitated by addition of 0.1 x volume of 3 M sodium acetate and 1 x volume of isopropanol at −20 °C, purified via a denaturing urea polyacrylamide gel (8 M urea, 1 × TBE, 19:1 acrylamide:bis-acrylamide) and eluted by “crush-and-soak” in 0.3 M sodium acetate pH 7.0 at 4 °C overnight while rotating. The RNAs were buffer exchanged to 150 mM NaCl, 50 mM Tris pH 7.0 and 1 mM TCEP in an Amicon® centrifugal filter unit and stored at –80 °C until used. RNAs used for OT experiments (*Ox40* full-length (fl), tandem ADE-CDE (ADE-CDE), ADE, and CDE) were transcribed with 5’ and 3’ overhangs to anneal with DNA handles (*SI Appendix*, Table S6). Note that the OT fl construct contained residues 1 to 140, omitting the flexible 3’ tail. For NMR, CD, and EMSA experiments, RNAs were snap-cooled prior use. Single-stranded (ss) 5’ and 3’ fragments of the ADE were obtained from Horizon and dissolved in the protein buffer (see above). The annealed duplex (ds) was formed by mixing the 5’ and 3’ halves in equimolar amounts, incubation at 95 °C for 5 min and a slow cool-down to room temperature on the bench.

### NMR Spectroscopy.

All NMR experiments were performed on Bruker Avance III Spectrometer operating at 700 and 950 MHz proton Larmor frequency equipped with triple-resonance cryogenic probeheads. For measurements and processing of spectra Topspin versions 3 and 4 were used. Spectra were analyzed with NMRFAM-Sparky 1.414 (47).

HNN-COSY (30) experiments were recorded as a BEST-TROSY version on a 275 µM ADE sample for the apo state and in presence of a twofold molar excess (550 µM) of coreROQ at 283 K. The transfer time was 30 msec, and 384 indirect points were collected for the apo RNA and 256 for the complex. H-bond J-couplings were calculated according to

[1] |h2JNN|=−INaINdatan2πΔ

where I denotes the intensity of the H-bond donor (Nd) or acceptor (Na) and 2∆ is the COSY transfer time.

^1^H,^15^N-HSQC spectra were recorded on uniformly labeled ^15^N apo coreROQ and extROQ proteins and in presence of a 1.2-fold molar excess of wt or mirrored ADE and a threefold molar excess of ss or dsRNAs. The protein concentration was 80 µM except for the complex with the mirrored ADE, where 20 µM of protein was used. A ^1^H,^1^H-NOESY of the mirrored ADE was recorded at 283 K on a 300 µM sample with 416 indirect points and a mixing time of 250 msec.

### OT Measurements.

#### DNA handle preparation and hybridization reaction.

Biotin and digoxigenin-functionalized handles were PCR-amplified separately using a lambda-phage template DNA, forward primers with biotin or digoxigenin modifications, and backward primers introducing ssDNA overhangs (all sequences are listed in *SI Appendix*, Table S6). Primers were supplied by Biomers. Water was ultrafiltrated by Sartorius arium pro UF. Other components for the PCR were ordered from NEB. First, 300 µl of reaction mix (1x Thermopol buffer, 0.2 mM dNTPs, 6 µl lambda-phage DNA, and 1 µM forward (biotin/digoxigenin) and backward primers, 1 µl Taq polymerase) were set up and split equally (50 µl) into six PCR tubes and run in the peqSTAR thermocycler from PEQLAB (for thermal protocol see *SI Appendix*, Table S7). PCR products were purified using the Monarch PCR & DNA Cleanup Kit (NEB), using the hybridization buffer [300 mM NaCl, 50 mM Tris (pH = 9.0), 20 mM EDTA (pH = 8.0)] for elution. Then, the quality of isolated products was verified via agarose gel electrophoresis (1.5%) using TAE buffer (40 mM Tris, 20 mM Acetic acid, 1 mM EDTA).

Assembly of the entire construct used for measurement, comprising sample RNA (same procedure and molar concentration was used for every *Ox40* 3’ UTR element construct), adapter, and handles, was performed in two separate hybridization steps. Biotin-functionalized handles were brought to reaction with the ssDNA adapter, while digoxigenin handles were directly attached to the sample RNA. Handle DNA was diluted to a final concentration of 0.071 µM and mixed with the molecule to anneal to in a 1:1 molar ratio in the hybridization buffer, then hybridized (for thermal protocols see *SI Appendix*, Tables S8 and S9). In a second hybridization reaction, the products of the first step were joined and incubated over night at room temperature. Final products were quality controlled via agarose gel electrophoresis (1.5%, TAE buffer), and stored at −80 °C. In the end, the complete dumbbell construct is formed when the hybridized construct is connected to Digoxigenin and Streptavidin functionalized micron-sized silica beads that are trapped in a focused laser beam (Fig. 1*B*).

#### Measurement preparation.

To avoid simultaneous binding of multiple molecules to the same bead pair, assembled constructs were diluted to around 0.1 nM and incubated at room temperature for 10 min with micron-sized streptavidin-coated (SV) beads (Bangs Laboratories, Inc.) in HS buffer (20 mM MgCl2, 300 mM KCl, 50 mM HEPES). After incubation, this solution was diluted into 300 µl of HS buffer. In similar manner, anti-digoxigenin-coated (AD) beads were diluted to identical concentration in HS buffer with the same scavenger system. In parallel, 500 µl of each running buffer for the three flow channels were prepared by addition of the scavenger system [final concentrations: 26 U/ml glucose oxidase (SIGMA-ALDRICH), 17,000 U/ml catalase (SERVA), and 0.65% glucose (SIGMA-ALDRICH)] to the corresponding measurement buffer. A commercial microfluidic chip [C-Trap® Optical Tweezers—Fluorescence and Label-free Microscopy (LUMICKS)] was used that contains two bead channels and additionally allows maintenance of three different phases during measurement, separated by laminar flow (*SI Appendix*, Fig. S5). For a detailed description of the standard experimental workflow with this system, see *SI Appendix*, *Supplementary Methods*. All measurements were performed using a trap stiffness between 0.25 pN/nm and 0.40 pN/nm, and at temperatures of ~ 25 °C. Data were downsampled from the initial sampling rate of 78.125 kHz by a factor of 3 before analysis.

### Rate Determination.

To extract on- and off-rates of coreROQ or extROQ to the ADE, passive-mode traces were HMM assigned (32) to ROQ-bound and unbound states. Off-rates can be determined directly by taking the inverse of the average lifetime of bound states, while calculation of on-rates requires to take into account the folding distribution at the given force (see *SI Appendix*, *Supplementary Methods* for more details). For the on and off-rates in Fig. 3 *B* and *C* and *SI Appendix*, Fig. S8 *B* and *C* one analyzed passive mode step resulted in one data point. Furthermore, we used the number of analyzed passive mode steps as primary unit “N” of the analysis in *SI Appendix*, Figs. S7 *B* and *C* and S11 *B* and *F* and Table S3–S5. In *SI Appendix*, Table S2 “N” refers to the number of analyzed force-extension cycles and in *SI Appendix*, Fig. S4 to the number of state occurrences (for more details, see *SI Appendix*, Table S10).

### Circular Dichroism Spectroscopy.

CD spectra were recorded on a JASCO J-810 spectrometer in the protein buffer from 180 to 320 nm (*SI Appendix*, Fig. S9*B*). 20 µM ADE RNA were measured in presence of 0 and 40 µM of coreROQ or extROQ. For melting curves, the change in the CD signal at 263 nm was monitored from 5 to 95 °C with a temperature gradient of 1 °C/min for 20 µM apo ADE RNA or in presence of 40 µM of the respective protein. Curve fitting for separated T_m_s was done in Origin with DoubleBoltzmann fits. Apo protein melting curves were recorded at 220 nm of 10 µM extROQ and 40 µM coreROQ samples. The same protein concentrations were used for apo protein CD spectra for comparison to RNP spectra.

### Electrophoretic Mobility Shift Assay.

For EMSAs RNAs were fluorescently 5’-labeled with fluorescein as described before (8). EMSAs were performed according to (48). Briefly, 3 nM of ADE RNA were incubated with 0, 100, 200, 400, 700, 1,000, 2,000, 3,000, 5,000, and 20,000 nM of coreROQ or extROQ. Complexes were run on a native 6% polyacrylamide gel at 4 °C for 1 h. Gels were imaged with a ChemiDoc Imager (BioRad). Quantification of gel bands was performed with ImageJ and K_D_s were calculated as averages plus SD from triplicates fit individually with a Hill1 fit in Origin.

### Hydroxyl Radical Probing of RNA.

60 pmol of fluorescently labeled ADE RNA apo or in presence of a threefold molar excess of coreROQ or extROQ were rapidly mixed with 1 mM Fe(II), 2 mM EDTA, 1 mM sodium ascorbate and 0.06% (w/w) H_2_O_2_ according to (49). After 3 min of incubation, samples were quenched with 10 mM thiourea and loaded onto a denaturing 10% polyacrylamide gel (8). For imaging a Typhoon 9400 Variable Mode Imager (GE) was used and gray scale intensity profiles for all lanes were extracted with ImageJ. These profiles were plotted against the nucleotide residue number obtained from the alkaline ladder. Alkaline and T1 ladders were generated as described before (8).

## Supplementary Material

Appendix 01 (PDF)

## Data Availability

All study data are included in the article and/or *SI Appendix*. SMFS raw output data from Bluelake 2.4.2 (Lumicks) with analysis instructions using the licensed software Igor Pro 8.0 (Wavemetrics) are available at https://doi.org/10.6084/m9.figshare.28690193.v1 (50). Raw data for CD spectroscopy, NMR, chemical RNA probing and the EMSAs are available at https://doi.org/10.6084/m9.figshare.28692116 (51).
